# Porokeratosis simulating Bowen’s disease on dermoscopy*

**DOI:** 10.1590/abd1806-4841.20164479

**Published:** 2016

**Authors:** Alzinira Sousa Herênio, Silvana Maria de Morais Cavalcanti, Emmanuel Rodrigues de França, Clarissa Marques Maranhão, Eliane Ruth Barbosa de Alencar

**Affiliations:** 1 Universidade de São Paulo (USP) – São Paulo (SP), Brazil; 2 Universidade de Pernambuco (UPE) – Recife (PE), Brazil

**Keywords:** Dermoscopy, Bowen’s disease, Lymphedema, Porokeratosis

## Abstract

Porokeratosis is a disorder of epidermal keratinization characterized by the presence of annular hyperkeratotic plaques. Its etiopathogenesis is not yet fully understood, but a relationship with immunosuppression has been reported. Dermoscopic examination revealed a classic yellowish-white ring-like structure that resembled “volcanic crater contour” – the so-called cornoid lamella. We describe a case of porokeratosis in a female patient with chronic lymphedema, which was similar to Bowen’s disease due to the many glomerular vessels seen on clinical examination and dermoscopy.

## INTRODUCTION

Porokeratosis is a disorder of keratinization characterized by annular hyperkeratotic plaques with raised borders.^1^ At least five clinical variants are reported in the literature, and all of them share one characteristic, the cornoid lamella, which is a thin column of closely stacked parakeratotic cells in an area of epidermal invagination.^1^ An association between porokeratosis and malignancy has been described, including Bowen's disease (BD). Immunosuppression is a risk factor for malignancy.^2^

We report a case of porokeratosis in a female patient with chronic lymphedema, which was similar to BD on clinical examination and dermoscopy. BD is an important differential diagnosis in cases of lesions with a glomerular pattern under dermoscopy.

## CASE REPORT

**Figure 1:**
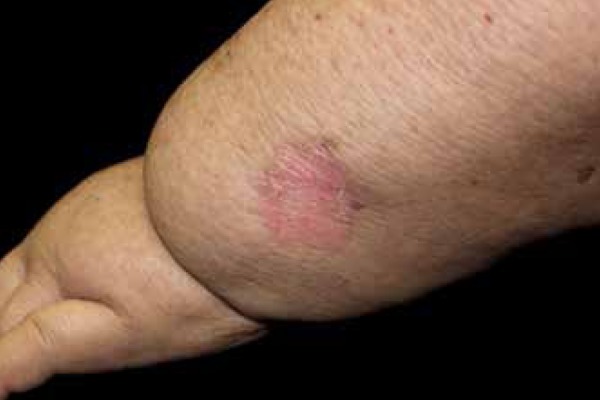
Lymphedema on the left forearm with an erythematous plaque lesion and keratotic border

**Figure 2:**
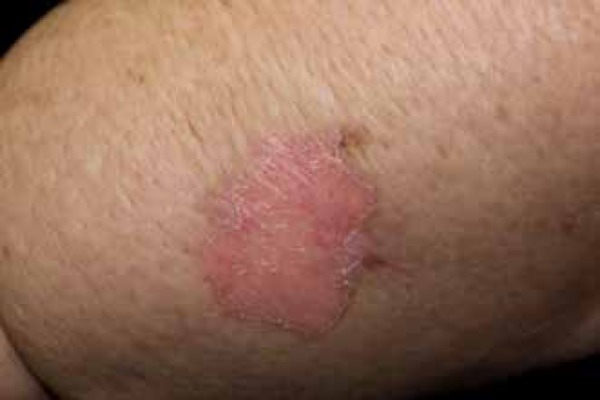
Edematous skin with an erythematous plaque lesion and keratotic border on the left forearm

**Figure 3:**
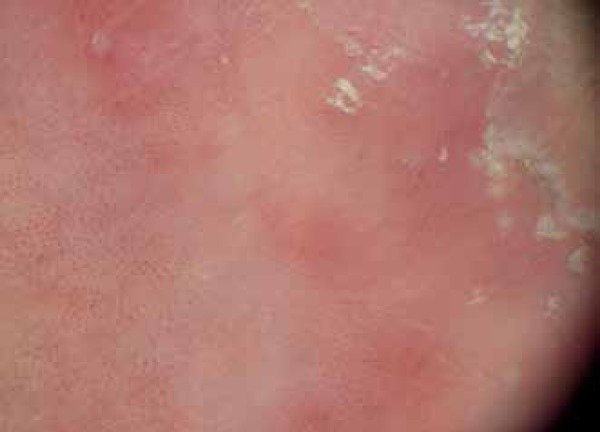
Dermoscopy: presence of abundant glomerular vessels throughout the lesion with an erythematous background and keratotic border

**Figure 4:**
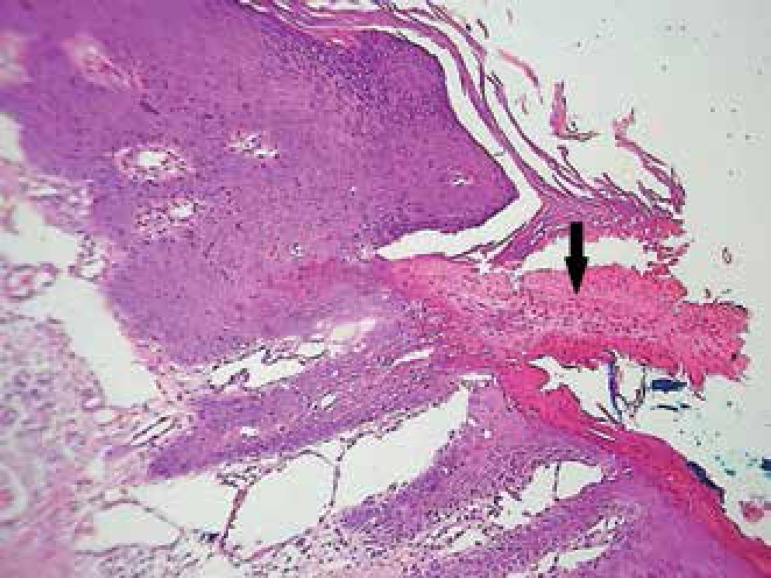
Histopathology: cornoid lamella (black arrow) and absence of cell atypia (HE)

A 74-year-old female patient reported a history of an asymptomatic lesion on her left forearm in the last three years. After mastectomy, the patient presented with chronic lymphedema on the affected limb with ipsilateral axillary dissection. The malignant breast tumor was treated with subsequent radiotherapy sessions. Clinical examination revealed a well-defined erythematous plaque with raised borders of approximately 3 cm in diameter on her left forearm (Figures 1 and 2). With dermoscopy we observed homogeneous glomerular vessels throughout the lesion with an erythematous background and keratotic border (Figure 3). BD was a possible diagnosis. Histopathological examination revealed the presence of a cornoid lamella and the absence of cell atypia, thus confirming the diagnosis of porokeratosis (Figure 4).

## DISCUSSION

Porokeratosis was first described by Mibelli and Respighi in 1893.3 It is a disorder of keratinization, and at least five variants are recognized, as follows: 1) porokeratosis of Mibelli; 2) disseminated superficial porokeratosis; 3) disseminated superficial actinic porokeratosis (DSAP); 4) linear porokeratosis; and 5) porokeratosis palmaris et plantaris disseminata.^1^ The disease predominates in males, and the lower limbs are the sites most commonly affected in porokeratosis of Mibelli and DSAP.^4^ All these variants are associated with the presence of a cornoid lamella (a column of keratotic cells in an area of epidermal invagination seen through histopathology). Porokeratosis may be considered a premalignant lesion, with a risk of change at around 7.5%.^2^

Although the clinical presence of a single lesion associated with immunosuppression suggested the diagnosis of porokeratosis of Mibelli, it is worth mentioning that this variant is epidemiologically more common in children and male patients, different from our patient, who is an elderly woman.^5^

Dermoscopy is a non-invasive diagnostic method which uses a magnifying lens combined with immersion (use of gel, oil or other liquid) or with polarized light filters to reduce refraction. It allows the visualization of pigmented and vascular structures extending from the stratum corneum to the papillary dermis.^6^ Under dermoscopy, porokeratosis typically reveals a yellowish-white ridge-like structure showing a pale pink area of central atrophy that resembles volcanic craters. Rare red globular structures may be present, differently from what is observed in squamous cell carcinoma *in situ*, in which these structures are abundant.^7^ Under dermoscopy our patient presented with a keratotic lesion showing many glomerular vessels, compatible with BD symptoms. BD shows a 98% probability of squamous cell carcinoma *in situ* when glomerular or punctate vessels associated with hyperkeratosis are present.^8^ Histopathology was paramount to rule out malignancy due to the absence of atypia and the presence of a cornoid lamella, which led to the diagnosis of porokeratosis.

Porokeratosis has been reported in immunocompromised subjects such as patients who have undergone transplant, those with hematological neoplasms and those who have undergone chemotherapy, previous irradiation, and corticotherapy.^9^ This is due to the growth of abnormal epidermal clones, a fact confirmed by lesion improvement after the immunosuppressing factor ceases to exist.^10^ Because lymphedema causes or results from local immunosuppression, it is also a risk factor for the development of porokeratosis. The case herein described involves the presence of a lymphedema on the upper left limb after mastectomy with ipsilateral axillary dissection and subsequent radiotherapy sessions to treat a malignant breast tumor, which is a known risk factor for porokeratosis.

Hence, we highlight the importance of this diagnosis in immunosuppressed patients, even if the lesion may suggest neoplastic disease upon clinical examination or dermoscopy. Histopathology is essential to rule out malignancy and confirm possibly unexpected differential diagnoses.
